# *StatModPredict*: A user-friendly R-Shiny interface for fitting and forecasting with statistical models

**DOI:** 10.1371/journal.pone.0329791

**Published:** 2025-08-07

**Authors:** Amanda Bleichrodt, Amelia Phan, Ruiyan Luo, Alexander Kirpich, Gerardo Chowell

**Affiliations:** 1 Department of Population Health Sciences, School of Public Health, Georgia State University, Atlanta, Georgia, United States of America; 2 Department of Applied Mathematics, Kyung Hee University, Yongin, Korea; National Chengchi University, TAIWAN

## Abstract

**Background:**

Many disciplines, such as public health, rely on statistical time series models for real-time and retrospective forecasting efforts; however, effectively implementing related methods often requires extensive programming knowledge. Therefore, such tools remain largely inaccessible to those with limited programming experience, including students training in modeling, as well as professionals and policymakers seeking to forecast an epidemic’s trajectory. To address the need for accessible and intuitive forecasting applications, we present *StatModPredict*, an R-Shiny dashboard for conducting robust forecasting analysis utilizing auto-regressive integrated moving average (ARIMA), generalized linear models (GLM), generalized additive models (GAM), and Meta’s Prophet model.

**Methods:**

*StatModPredict* supports robust real-time forecasting and retrospective model analysis, including fitting, forecasting, evaluation, visualization, and comparison of results from four popular models. After loading an incident time series data set into the interface, users can easily customize model parameters and forecasting options to obtain the desired output. Additionally, *StatModPredict* offers multiple editable figures for, but not limited to, the time series data, the forecasts, and model fit and forecast metrics. Users can also upload external forecasts produced elsewhere and evaluate their performance alongside the dashboard’s built-in models, thereby enabling direct comparisons. We provide a detailed demonstration of the dashboard’s features using publicly available annual HIV case data in the US. A video tutorial is available at https://www.youtube.com/watch?v=zgZOvqhvqw8.

**Conclusions:**

By eliminating programming barriers, *StatModPredict* facilitates exploration and use by students training in forecasting, as well as professionals and policymakers aiming to forecast epidemic trajectories. Additionally, the flexibility in the required input data structure and parameter specification process extends the application of *StatModPredict* to any discipline that employs time series data. By offering this open-source interface, we aim to broaden access to forecasting tools, promote hands-on learning, and foster contributions from users across disciplines.

## Introduction

Statistical and mathematical modeling have become critical public health resources for characterizing and forecasting the epidemic trajectory of various diseases on different spatial scales [1–5]. Common approaches range from traditional compartmental epidemic models (e.g., SEIR) [6–9] and machine learning algorithms [10–13] to more recent techniques, such as ensemble sub-epidemic frameworks [2,14,15], which are widely used in disease modeling and forecasting efforts. However, simple statistical methods, such as the auto-regressive integrated moving average (ARIMA) model, are appealing due to their minimal data requirements and utility as a benchmark for comparison against more complex analytical approaches.

Multiple statistical models for time series data, such as ARIMA, generalized linear regression models (GLM), generalized additive models (GAM), and Meta (Facebook’s) Prophet (Prophet) model, have become popular forecasting and benchmarking tools in disease modeling [2,15–23]. These models are popular due to their robustness in diverse contexts, requiring minimal data while allowing extensive customization to achieve optimal fit and forecast results. Nevertheless, their use often requires programming expertise. As a result, they are frequently inaccessible to students training in disease modeling and professionals with limited programming or mathematical backgrounds.

User-friendly graphical interfaces incorporating a range of public health modeling and forecasting methodologies have become increasingly accessible to the public. These tools span from interactive platforms for exploring existing forecasts [24–32] to software designed to support users in conducting their own forecasting analyses [33–44]. For example, Liu et al. developed a web-based platform to explore Canada’s COVID-19 situation and included various visualizations and forecasts from multiple modeling frameworks [24]. Additionally, the Delphi Group, based at Carnegie Mellon University, produced many interactive tools for manipulating and visualizing COVID-19 forecasts at the state and national levels in the United States [27,28]. While such interfaces provide accessible insights into specific contexts, they rarely facilitate end-to-end workflows for real-time and retrospective model fitting and forecasting efforts.

Existing applications that support customizable forecasting analysis are often restricted to a single modeling framework or process of interest, provide limited visualization tools, or are specified for a single temporal resolution (i.e., weekly) [33–36,42–44]. Additionally, they usually require enrollment in a paid subscription program to access all available features [33,34,37–40]. For example, the *Forecast* feature of Looker, a Google Cloud dashboard application, utilizes its own version of an automatic ARIMA algorithm (*AutoARIMA*) to produce forecasts and related visualizations. However, model fit and forecast performance metrics are not readily available to users, and only a single ARIMA modeling framework is included [36]. Streamlit Prophet provides additional details related to model fit and forecast performance, but it is limited to the Prophet model and does not facilitate model-to-model comparison [41]. While robust open-source tools exist, such as *predictoR()* [32] and *Greymodels()* [31], they do not currently support comparison to externally generated models and offer only limited parameter customization or extension beyond their predefined modeling frameworks.

Given the importance of statistical and mathematical modeling and forecasting in public health contexts, there is a need for comprehensive and accessible applications that require no previous programming experience. Such software can facilitate the training of students focused on areas of study such as public health or mathematical biology and can quickly provide readily accessible forecasting methods to interested professionals. Therefore, we present *StatModPredict*, a novel, user-friendly, R-Shiny dashboard for fitting and forecasting time series data using four established statistical models: 1) ARIMA, 2) GLM, 3) GAM, and 4) Prophet. *StatModPredict* is built around the well-tested *auto.arima()* [45*]*, glm()** [46] and *glm.nb()* [47], *gam()* [48], and *prophet()* [49] R-packages, each of which supports flexible parameter specification and subsequent forecasting.

The dashboard also enables users to obtain commonly employed model fit and forecast performance metrics for each included statistical model. Additionally, users can incorporate previously and externally (i.e., using some other software or code) conducted forecasts and performance metrics to perform direct comparisons against the models available within the dashboard. By eliminating the requirement for prior programming and coding experience, *StatModPredict* addresses the need for an open-source, graphical interface for complex real-time and retrospective forecasting analyses such as those described in Refs. [2,4,5,15,19,21,50] and as applicable in other fields that utilize statistical time series models.

To demonstrate the utility of *StatModPredict*, we begin with an overview of the features and underlying methodology applied in the dashboard, including data preparation, its implementation, and the statistical methods. We then present a step-by-step tutorial using publicly available yearly Human Immunodeficiency Virus (HIV) epidemic data from the United States to illustrate the dashboard’s real-world applications. A video tutorial demonstrating the *StatModPredict* toolbox on the same HIV data set can be found online at https://www.youtube.com/watch?v=zgZOvqhvqw8&t=4s [51].

## Materials and methods

Before launching the dashboard, users must first download the latest versions of R from CRAN [52] and RStudio from Posit [53]. While *StatModPredict* is housed within the RStudio integrated development environment (IDE), no previous experience with R or RStudio is required to interact and access the full features of the application. Table 1 outlines the necessary system requirements and provides the link to the host repository.

**Table 1:** *StatModPredict* metadata.

Require Software	R (>= 4.3), RStudio (>= 2024.09.0 Build 375)
**Compilation requirements** ^a^	*pacman, MASS, shiny, shinydashboard, shinyWidgets, bslib, plotly, anytime, shinyalert, shinyjs, shinybusy, editData, shinyBS, DT, stringr, tidyverse, forstringr, mgcv, processx, ggpubr, forecast, prophet, zip, glue, shinyjqui, patchwork, ggplot2, zoo, gridExtra, viridis, qdapRegex, RColorBrewer, chron, lubridate*
**Permanent link to repository**	https://github.com/bleicham/StatModPredict [54]

Table 1 contains the software, versions, and packages needed to launch the *StatModPredict* R-Shiny application successfully. Additionally, it provides a link to the permanent repository, which includes all necessary functions, the user interface file, and tutorial materials.

^a^Upon initial compilation of the dashboard, it checks if the required packages are downloaded. If they are not, R will proceed to install the packages for the given session. During this process, pop-up messages may appear asking if the user would like to compile the package. To successfully utilize all dashboard features, the user must select “yes” and ensure that all required packages are added.

To launch *StatModPredict*, users should first open the *StatModPredict* R-project and then the ‘app.R’ file. Once the user interface file has been successfully loaded (‘app.R’), the dashboard will appear after the ‘Run App’ button has been clicked. Below describes the structure of each of the pages included within *StatModPredict*: 1) *About*, 2) *Forecasting*, 3) *Metrics*, and 4) *Model Comparison*.

### About page

Upon initially loading the application, users will see a “Welcome” message and be directed to the *About* page. This page provides an overview of the methods employed within the dashboard, including model specification, forecasting, and details of the available evaluation statistics. Additionally, as some features require nuanced file specifications, we provide step-by-step instructions for preparing files in the correct format within this page.

### Forecasting page

**Fig 1:**
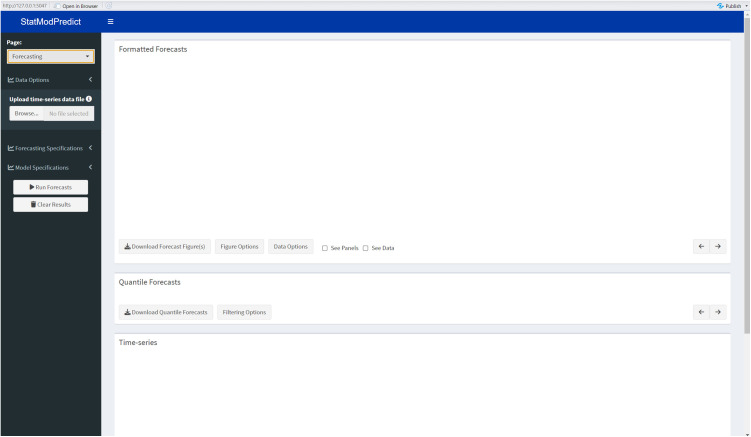
A screenshot of the *Forecasting* page after initially loading the dashboard. Users primarily interact with the dashboard’s sidebar, where details related to the data, forecasting specifications, and all model parameters must be entered. Once selected, the user can then click “Run Forecasts” to obtain output. The *StatModPredict* dashboard was developed using R-Shiny.

The *Forecasting* page serves as the starting point for utilizing the *StatModPredict* dashboard (Fig 1), including loading the time series data and specifying the model fitting and forecasting parameters. After selecting all necessary options and clicking the “Run Forecasts” button, the resulting models’ fits, forecasts, and appropriate visualizations will be displayed on this page. Note that if options have not been specified and processed, the remaining dashboard features (i.e., model metrics and model comparison) will not be accessible. Additional details regarding the required inputs and available outputs are provided below.

#### Input data.

The dashboard allows users to utilize any incident time series data, provided it follows the correct formatting guidelines and is a *.csv file. Users must structure their data in a “wide” format, where the first column corresponds to the time series dates (i.e., daily, weekly, yearly, or time index), and the remaining columns include the counts of the process of interest for each location or group at each time point. All columns must have headers without dashes and any other special characters in their names; however, there is no restriction on the file’s name. The dashboard requires an evenly spaced time series without any missing or repeated dates.

**Fig 2:**
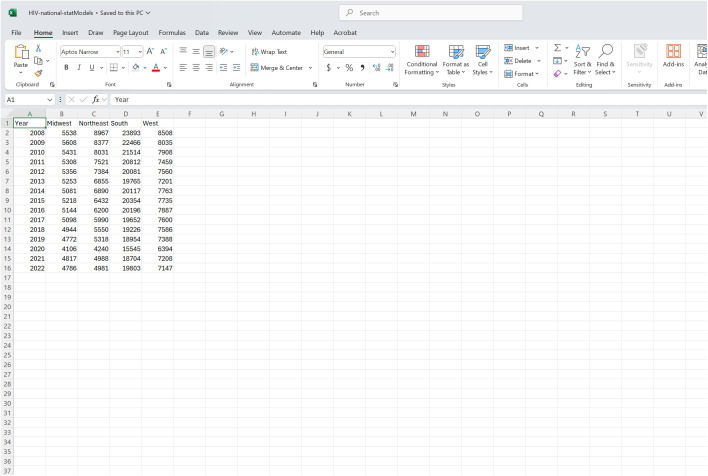
An example of the data structure needed for the dashboard. The data shown above corresponds to yearly incident HIV diagnoses in the United States obtained from the publicly available Centers for Disease Control and Prevention (CDC) *AtlasPlus* dashboard [55]. As can be seen, the first column corresponds to the temporal resolution (i.e., years). The remaining columns contain the counts of the phenomena of interest (i.e., incident HIV diagnoses) for each group or location.

When working with daily or weekly data, years must be formatted with four digits (YYYY); any conventional format can be used for the month and day. However, if employing yearly data, only a four-digit year can be used (YYYY). If a user elects to proceed with time indexes rather than dates, the first row of data corresponds to a time index of 1. Regardless of the temporal resolution, records over time should be presented from top to bottom of the data set (i.e., from oldest to newest). Once loaded, the dashboard will automatically determine if the input time series contains daily, weekly, yearly, or time index data. Fig 2 illustrates an example of the proper file structure with annual data, and other temporal-type time series structures can be found in the GitHub repository [54] listed in Table 1.

There are no programming-related restrictions on the type of time series data used within *StatModPredict*, given that it follows the format discussed above. However, it is essential to consider the assumptions of the selected models and their characteristics (i.e., error distributions) in the context of the process of interest before engaging in model analysis. For example, when working with Poisson or negative binomial distributions, only count data can be used. Additional guidance on data-specific recommendations for each available model type is provided below.

#### Data options.

After uploading the time series via the “Upload time-series data file” button, users can further pre-process their data, depending on its temporal resolution. For example, when working with daily time steps, users can apply a rolling average to reduce “noise” in the data [15]. More specifically, surveillance data reported in real-time often contain spurious fluctuations due to uncontrollable effects (e.g., reporting delays, weekend effects). Therefore, we provide the option to “smooth” the loaded noisy data using the *rollmean()* function from the “zoo” package [56]. If applicable to the loaded data, the user will see a numeric input appear allowing them to specify the desired length of the rolling average.

We only provide this smoothing option for daily time series, as noise is often minimal in weekly and yearly data. Additionally, the option is not available for the Prophet model, and some model fit metrics (i.e., Mean Squared Error (MSE), Mean Absolute Error (MAE), Prediction Interval (PI) Coverage, and Weighted Interval Scores (WIS)) are not calculated when smoothing is applied. Due to the assumptions of the Poisson and negative binomial distributions available for the GLMs and GAMs, the produced smoothed average is rounded to the nearest integer before model calibration. The rounding does not occur when the Gaussian/normal distribution is assumed.

The remaining data filtering option (i.e., “Location/Group”) auto-populates based on the column names of the time series file.

#### Forecasting specifications.

*StatModPredict* enables both retrospective and prospective predictions of processes of interest, which are indicated by the selected “Forecast Date(s)”. The “Forecast Date(s)” parameter, located within the sidebar, refers to the last time point of data used to calibrate the model. It can be thought of as the date on which the forecast is (real-time forecasting) or would have been (retrospective forecasting) “conducted”. For example, if we used the time series presented in Fig 2 and selected a forecast date of 2022, the chosen model would be calibrated with data through 2022. The dates available to users correspond to the first column included in the original input data. Therefore, the forecast dates that would be available, based on Fig 2, would be 2008–2022. Additionally, users can select multiple forecast dates at a time; thus, if multiple are chosen, multiple forecasts will be conducted.

The “Calibration Period Length” corresponds to the number of time points used to “calibrate” the model, ending on the indexed forecast date. Therefore, the available calibration period lengths depend on the number of time points available before, and including, the first selected forecast date. For example, using the data presented in Fig 2, if the user chooses a forecast date of 2008, no earlier years are available. Therefore, the only possible calibration period length is one (i.e., the forecast date). However, if the user selects 2022, the available lengths range from one (i.e., the forecast date) through 16 (i.e., the start of the data). If the user selects multiple dates, the largest available calibration period length is based on the earliest forecast date chosen.

Additionally, the start of the calibration period is not fixed to the first date available; rather, it depends on the selected calibration period length. For example, if the user chooses a forecast date of 2022 and a calibration period length of 2, and the first available year of data is 2008, the calibration period would range from 2020 through 2022. Finally, like forecast period dates, multiple calibration period lengths can be selected.

“Forecast Horizon” refers to the number of future time points the user would like to predict after the forecast date.

#### Modeling specifications.

**Fig 3:**
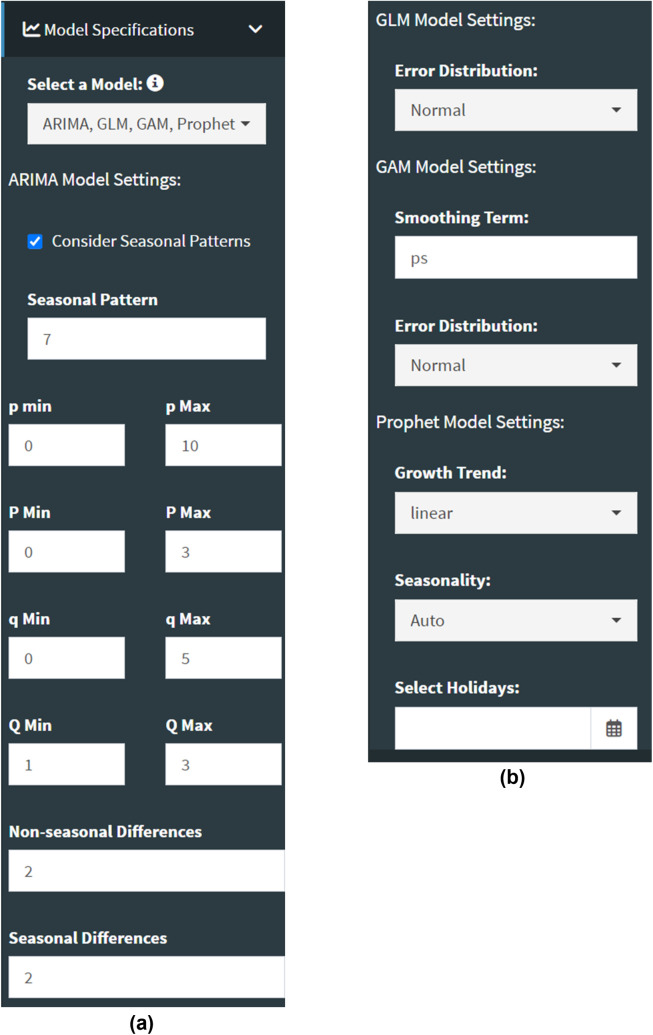
A screenshot of the available ARIMA (a), GLM (b), GAM (b), and Prophet (b) specifications. The options shown above are the default dashboard model settings. When data are loaded, an additional setting is available for GAMs to customize the number of basis functions, *k*, considered during the model fitting process. The selected calibration period lengths determine the default value for *k*, although it remains customizable if the user prefers to choose their own.

*StatModPredict*’s primary function is to facilitate fitting standard statistical models (i.e., ARIMA, GLM, GAM, and Prophet) to time series and producing subsequent forecasts. We employ multiple established R functions, *auto.arima()* [45], *glm()* [46], *glm.nb()* [47], *gam()* [48], and *prophet()* [49], to fit the selected models to the user’s data, with additional error structure assumptions (i.e., Gaussian/normal, Poisson, and negative binomial) and model parameter specifications, when applicable. All available model-specific parameters correspond to the built-in options provided by each utilized function (Fig 3).

**Auto-regressive integrated moving average (ARIMA)**

Autoregressive integrated moving average (ARIMA) models are a commonly employed approach to time series forecasting [2,15,17,21–23,50,57–61], primarily focused on describing the autocorrelations within the data [62]. ARIMA models consist of three parts: 1) auto-regressive models, 2) moving average models, and 3) integration, which aids in increasing data stability. Additional details regarding the ARIMA model and its associated components can be found in Ref. [62].

*Non-seasonal ARIMA models (ARIMA)*

Auto-regressive models of order *p,* or AR(p), involve using a linear combination of past values to predict the current value of the time series. The models can capture various time series patterns and thus are robust to different processes of interest. However, they are well-suited for modeling continuous, non-seasonal univariate time series data. The AR(p) model is given by [62]:


$$y_{t}=c+\phi_{1}y_{t-1}+\phi_{2}y_{t-2}+\ldots +\phi_{p}y_{t-p}+\varepsilon_{t}$$ (1)

where $\varepsilon_{t}$ is white noise, *c* is a constant, $\begin{array}{c} {\phi_{1},\ldots ,\phi }_{p} \end{array}$ are the model parameters, and $y_{t}$ is the value of the time series at time t.

Moving average models of order q, or MA(q), relate the current value of the time series with the past errors [62]:


$$y_{t}=c+\varepsilon_{t}+\theta_{1}\varepsilon_{t-1}+\theta_{2}\varepsilon_{t-2}+\ldots +\theta_{q}\varepsilon_{t-q}.$$ (2)

Like Eq. 1, $\varepsilon_{t}\;$ is a white noise process with mean zero and variance $\sigma^{2}$, *c* is a constant, $\theta_{q}$ controls the time series pattern, and $\varepsilon_{t-q}$ are the lagged error terms.

When working with a stationary stochastic process whose joint probability distribution does not change with time, both the AR(p) and MA(q) models can be consolidated into an ARMA model [62]:


$$y_{t}-\phi_{1}y_{t-1}-\phi_{2}y_{t-2}-\phi_{p}y_{t-p}=c+\varepsilon_{t}+\theta_{1}\varepsilon_{t-1}+\theta_{2}\varepsilon_{t-2}+\theta_{q}\varepsilon_{t-q}.$$ (3)

where the notation follows that described for (Eq. 1) and (Eq. 2). However, when the data shows evidence of non-stationarity and the mean changes over time (i.e., trend), ARIMA (*p,q,d*) models can be employed as they first involve a differencing (*d*) step which is used to remove certain trends. The difference between consecutive observations is computed to differentiate the data, such as $y_{t}-y_{t-1}$. Then, the ARMA model can be applied to the data after differencing. The ARIMA model is given by [62]:


$$\left(1{-\phi }_{1}B-\cdots -\phi_{p}B^{p}\right)(1-B{)}^{d}y_{t}=c+(1+\theta_{1}B+\cdots +\theta_{q}B^{q})\;\varepsilon_{t}$$ (4)

where $(1{-\phi }_{1}B-\cdots -\phi_{p}B^{p})y_{t}$ corresponds to the AR(p) component, B represents the backshift operator, $(1-B{)}^{d}y_{t}$ corresponds to differencing the timeseries data *d* times, and the MA(q) component is given by $c+(1+\theta_{1}B+\cdots +\theta_{q}B^{q}varepsilon_{t}$. Finally, as in (Eq. 1) – (Eq. 3), $\varepsilon_{t}$ is the white noise process in the model.

*Seasonal ARIMA models (SARIMA)*

The ARIMA model can also capture various seasonal data patterns, with only a slight adjustment to (Eq. 4) and the process discussed above. To form a seasonal ARIMA model (SARIMA), we include additional parameters, (P, D, Q)m, where m represents the seasonal period [62]. Therefore, (Eq. 4) now becomes [62]:


$$\begin{array}{cc} & \left(1{-\phi }_{1}B-\cdots -\phi_{p}B^{p}\right){\left(1{-\Phi }_{1}B^{m}-\cdots -\Phi_{P}B^{mP}\right)(1-B)}^{d}{{\left(1-B^{m}\right)}^{D}y}_{t}\\ & =c+\left(1+\theta_{1}B+\cdots +\theta_{q}B^{q}\right)\left(1+\Theta_{1}B^{m}+\cdots +\Theta_{Q}B^{mQ}\right)\varepsilon_{t}. \end{array}$$ (5)

The AR(p), MA(q), and differencing terms are interpreted identically to (Eq. 4). The m parameter denotes the seasonal period, $1{-\Phi }_{1}B^{m}-\cdots -\Phi_{P}B^{mP}$ characterizes the seasonal AR(P) part, ${{\left(1-B^{m}\right)}^{D}y}_{t}$ indicates the seasonal differencing and the seasonal MA(Q) polynomial is given by $1+\Theta_{1}B^{m}+\cdots +\Theta_{Q}B^{mQ}$. $\varepsilon_{t}$ is the white noise process in the model [62].

*Software specifications*

We employ the *auto.arima()* function from the “forecast” package to automatically select the order values of the ARIMA and SARIMA models based on the specified data and parameter ranges. Additional details regarding the function can be found in Ref. [45]. To provide flexibility in model specification, users can choose values for each of the parameter ranges for the non-seasonal (*p, q, d*) and seasonal (*P, Q, D)m* ARIMA models. We use the *forecast()* function from the “forecast” package to produce all estimates, assuming the default settings discussed in Ref. [63].

**Generalized linear models (GLM)**

Generalized linear models (GLMs) extend linear regression models to allow for multiple types of assumed distributions (i.e., Poisson, negative binomial, Gaussian/normal). Therefore, users can apply the included models to both continuous (Gaussian/normal) and count (Poisson/negative binomial) time series data. The model contains three components: a random or stochastic component, a systematic component, and a link function [64]. As applied in this toolbox, a GLM is structured as follows [64]:


$$y_{t}\sim EF\left(\mu_{t},\psi \right),\;g\left(\mu_{t}\right)=\beta_{0}+\beta_{1}t.$$ (6)

The random component identifies the response variable, $y_{t}$, and its associated probability distribution $EF\left(\mu_{t},\;\psi \right)$, a distribution in the exponential family with mean $\mu_{t}$ and dispersion parameter ψ [64]. As applied in the toolbox, $y_{t}$ corresponds to the time series data values and can be continuous or discrete counts. Together, the single predictor,  t*,* and the intercept $\beta_{0}$ compose the systematic component of the GLM [64]. Here, we assume a simple GLM with only one predictor,  t, the time components of the original data (i.e., dates). Finally, the link component (g(·)) is responsible for the robust nature of the GLM framework to other error structure types. Specifically, the “link” designates a function connecting $\mu_{t}$ and the linear predictor, *t* [64]*.*

As applied in *StatModPredict*, we utilize three possible distributions: Gaussian/normal, Poisson, and negative binomial.

*Gaussian/normal:* The link function for the normal distribution is the identity function. Therefore, when the user selects the normal distribution for the GLM, the given model is the equivalent of a simple linear regression model [64].

*Poisson:* Poisson regression is a form of GLM that assumes a Poisson distribution, which is used to model counts and has the property of $\sigma^{2}=\mu$. Further details regarding the distribution can be found in Ref. [65]. As applied in the toolbox, a GLM assuming a Poisson-distributed outcome applies a logarithm link; thus, it is often referred to as “log-linear”. Using (Eq. 6), the application of the Poisson link is as follows [66]:


$$ln\left(\mu_{t}\right)=\beta_{0}+\beta_{1}t.$$ (7)

The general function, g(·) introduced in (Eq. 6) is now given as ln(·) to show the specific function used within Poisson regression.

*Negative binomial:* Negative binomial regression, like Poisson regression, requires count data. However, negative binomial models are more robust to over-dispersed data as they allow the variance to exceed the mean. For example, the quadratic mean-variance relationship is given by $\sigma^{2}=\;\mu \;+\mu^{2}/\;\psi$. Additional details regarding the estimation of ψ can be found in Ref. [67], and the link function is identical to that of the Poisson distribution.

*Software specifications*

We employ the *glm()* function from the “forecast” package to conduct simple linear and Poisson regression [46], and the *glm.nb()* function provided by the “MASS” package when assuming a negative binomial distribution [47]. We utilize the *predict()* function from the “stats” package to obtain model forecasts and fits [68]. Users can select the underlying distribution assumption (i.e., *Normal*, *Poisson*, or *Negative Binomial*) during model customization. If either *Poisson* or *Negative Binomial* is selected, we exponentiate the resulting model fits and forecasts to return results in the original scale of the data. We use the default settings for each function, as provided in their respective documentation.

**Generalized additive models (GAM)**

Generalized additive models (GAM) extend GLMs to include non-linear data patterns while maintaining similar levels of explainability and simplicity [69]. For example, unlike (Eq. 6), GAMs include a sum of unknown smooth functions of some covariates [70]. Therefore, they are well-suited for time series with smooth, nonlinear trends. In *StatModPredict*, with time as the only covariate, the GAM, assuming normality, is as follows [69]:


$$y_{t}=\beta_{0}+f\;(t)+\in_{t}$$ (8)

where f(·) is an unknown smooth function of time and $\in_{t}\sim N(0,\;\sigma^{2})$ [65]. The Poisson and negative binomial distributions follow that discussed above (for GLM), and both distributions employ a log-link, resulting in:


$$\text{\{ln}(y{\}}_{t})=\beta_{0}+f(t).$$ (9)

Regardless of the selected distribution, the smooth function f(·) is represented using basis functions (i.e., building blocks for complex functions). The default setting employed within the dashboard uses basis splines or piecewise polynomial functions [48]. Specifically,


$$f\;(t)=\sum {\mathrm{\backslash nolimits}}_{k=1}^{K}\beta_{k}b_{k}(t),$$ (10)

where {$b_{k}(.)$} represent the basis functions, $\left\{\beta_{k}\right\}$ are the expansion coefficients to be estimated, and *K* is the number of basis functions [69]*.* The number of basis functions depends on the length of calibration data available for a given forecast date; however, this can be customized as needed. Users can also select the specific smoothing term from Ref. [71]. We set the default to a discrete penalty based on coefficients and then fit the model by solving a penalized least squares problem (i.e., *ps* in Ref. [71]). For any GAM employed within the toolbox, the generalized cross-validation (GCV) criterion selects the smoothness tuning parameter [48].

*Software specifications*

We employ the *gam()* function from the “mgcv” package for model fitting [48], and users can select the underlying distribution assumption (i.e., *Normal*, *Poisson*, or *Negative Binomial*) during model customization. If either *Poisson* or *Negative Binomial* is selected, we exponentiate the resulting model fits and forecasts to return results in the original scale of the data. Additionally, while the number of basis functions is auto-populated based on the length of the calibration period, users can specify the number they wish to employ in the calculation of the smoothing function (Eq. 10). Finally, users can also indicate a different smoothing term than the default *ps* option, selecting one of the options provided in Ref. [71]. Once the model has been fit, we utilize the *predict()* function from the “stats” package to obtain model forecasts and fits [68]. Except for the available specifications discussed here, we use the default settings for both functions (*gam()* and *predict()*) provided in their respective documentation.

**Meta (formerly Facebook’s) Prophet model (Prophet)**

Meta’s (formerly Facebook’s) Prophet model has been increasingly employed across multiple fields to forecast different processes of interest. It is particularly suited for robust, seasonal time series data and can handle missing data, shifts in seasonal trends, and outliers [17,18,23,50,60,61,72–75]. The model’s specification is similar to that of the GAM and is decomposable into three components (i.e., trend, seasonality, and holidays) and an error term [76]. Thus, the model has the following form with time as the only regressor [76]:


$$y(t)=g(t)+s(t)+h(t)+\in_{t}.$$ (11)

Here, g(t) represents the non-periodic component for modeling the time series trend. Periodic trends (e.g., seasonal changes) are captured by s(t), and h(t) is an irregular events component used for modeling irregular changes (e.g., holidays or similar events). The component $\in_{t}$ represents the normally distributed model errors at time t. The model, including change points, is estimated via a Bayesian-style approach [62]. Additional details regarding the model can be found in Refs. [49,76].

*Non-periodic component,* g(t)

Within *StatModPredict,* users can select from *linear* or *uniform* growth trends. When a user chooses the *linear* growth option, g(t) becomes a set of piecewise linear equations where the growth rate varies between change points [76]. Thus, the non-periodic component is given by [76]:


$$g(t)=\left(k+a(t{)}^{⊺}\delta \right)t+(m+a(t{)}^{⊺}\gamma )$$ (12)

where k is the growth rate, and m is an offset parameter. The Prophet model allows the growth rate to change at various time points or change points. Therefore, there are S automatically selected change points at time $s_{j}$, where j = 1, ..., S. δ  represents the vector of rate adjustments, where $\delta_{j}$ is the rate change at $s_{j}$ [76]. The rate at time t is represented by $k\;+\;a(t{)}^{⊺}\;\delta$, where [76]:


$$a_{j}(t)=\left\{ \;\;\;\begin{array}{c} 1,\;\;\;if\;t\geq s_{j}\\ 0,\;Otherwise. \end{array}\right.\;$$ (13)

m, the offset parameter is also adjusted with each change to k. Finally, to ensure a continuous function, the adjustment at each change point j is given by $\gamma =-s_{j}\delta_{j}$. As applied in the dashboard, the change points are automatically selected by setting a sparse prior on δ. Additional details regarding the change points can be found in Ref. [76]. If the user selects the “flat” growth option, g(t becomes a constant value [76].

*Periodic component,* s(t)

The seasonal component of the Prophet model, s(t), relies heavily on a Fourier series. Specifically, the model approximates seasonal effects with the following standard Fourier series [76]:


$$\begin{array}{c} s(t)=\sum {\mathrm{\backslash nolimits}}_{n=1}^{N}[a_{n}cos(\frac{2\pi nt}{P})+b_{n}sin(\frac{2\pi nt}{P})] \end{array}$$ (14)

where P is the time series’ period. N represents the number of sine and cosine pairs included, and $a_{n}$ and $b_{n}$ are the coefficients of the Fourier series. The model estimates the 2N parameters, $\beta ={\left[a_{1},b_{1},..,a_{N},\;b_{N}\right]}^{⊺}$ by building a matrix of seasonality vectors for historical and future data at time *t*. Additional details can be found at Ref. [76]. If a user selects *Daily* seasonality, N = 4. If *Weekly* seasonality is selected, N = 3, and N= 10 when *Yearly* seasonality is selected [77]. If the user selects *Auto* for seasonality, Prophet automatically enables daily, weekly, or yearly seasonal components based on the data’s frequency and span (e.g., weekly and annual for daily data). If *None* is selected, the s(t) term disappears from *t*he model [76].

*Irregular events component*, h(t)

The final component of the Prophet model, h(t), allows the user to consider the effect of major holidays during the model fitting and forecasting process. Specifically, users can specify dates to be treated as “holidays,” where the effect of each selected date is independent [76]. Therefore, the irregular events component is given by [76]:


$$h(t)=[1\left(t\in D_{1}\right),\ldots ,1\left(t\;\in D_{i}\right)kappa$$ (15)

where $D_{i}$ is the past and future dates for a given holiday, i, and **1** is an indicator representing if time t falls during holiday i [76]. Finally, κ corresponds to the change in the forecast, where $\kappa \;\sim \;Normal(0,\;v^{2})$ [76].

*Software specifications*

We employ the *prophet()* function from the “prophet” package for model fitting [49]. As discussed above, users can select either a *linear* or *flat* growth trend assumption during the model-fitting process. Additionally, users have multiple seasonality assumption options, with the default set to *Auto* [49]. Finally, suppose some holidays should be considered as part of the model fitting and forecasting process. In that case, users can select multiple holidays (i.e., dates) via the calendar in the sidebar panel. However, the dashboard defaults to considering no holidays or special dates. We utilize the *predict()* function from the “stats” package to obtain model forecasts and fitted values [68].

#### Forecasting output.

**Fig 4:**
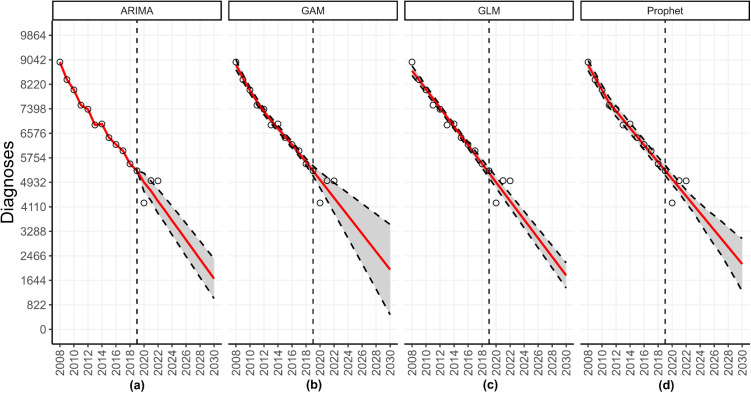
A panel of HIV forecasts for the Northeast United States obtained using data through 2022. This figure shows the forecasts produced for the Northeast United States utilizing yearly incident HIV diagnoses obtained from the publicly available Centers for Disease Control and Prevention (CDC) *AtlasPlus* dashboard [55], and assuming the default settings of *StatModPredict*. It can be obtained from the top box of the *Forecasting* page by clicking the “See Panels” option. For each model, the grey region corresponds to the selected prediction interval, the vertical dashed line is the forecast date (i.e., the last date used to calibrate the model), the red line is the estimated fit or forecast, and the open circles are the observed data. As can be seen, all four models forecast a decrease in the number of HIV diagnoses through 2030.

Once the user has indicated all options in the sidebar, the dashboard provides outputs related to the model fits, forecasts and time series data. For example, after fitting the specified models to the time series, the user can download customizable individual figures or panels of figures showing the model fit and forecasted trajectory grouped by location (or group) and forecast date (Fig 4).

The individual and panel forecast figures illustrate the model fit, forecast, and prediction interval overlaid with the available data. For example, as can be seen in Fig 4, the forecast extends past the range of available data. Therefore, some dates do not have observed data points plotted during the forecast period. However, if data become available after the forecast has been conducted, users can compare the model fit and forecasted trajectory against an updated data set. The updated data set must follow the same layout as the original data, including column names, and contain information for dates extending past the original time series. Users can also download the underlying tabular data used to plot the figures.

Other outputs include the “crude” forecast files containing 22 different quantiles, including the model fit and forecasted values for each selected model combination, location (or group), and calibration period. Finally, a customizable time series figure visualizing the trajectory of the data for the chosen locations is available for download. Additional examples of each available output can be found in the case study presented below.

### Model metrics page

After fitting the models to the data and producing forecasts, users can navigate to the *Model Metrics* page to obtain associated performance metrics. *StatModPredict* calculates various metrics, including MSE, MAE, WIS, PI coverage, Winkler scores, and skill scores, when applicable. We also provide the corrected Akaike Information Criterion (AICc), Akaike Information Criterion (AIC), and Bayesian Information Criterion (BIC) to evaluate the model fit of the ARIMA, GLM, and GAM, as well as the results of the Ljung-Box test for the ARIMA model. Additional details regarding the Ljung-Box test can be found in Ref. [62].

It is important to note that some model fit metrics cannot be calculated for the ARIMA-type models due to the structure of the *auto.arima()* function (MSE, MAE, WIS, and PI coverage), and in a limited capacity for the GLMs and GAMs when smoothing is applied. Additionally, forecast performance metrics will only be provided if observed data are available for the duration of the forecasted period. If updated data become available later, users can upload the new data to compare against the previously created forecasts. However, the latest data must extend past what was used to produce the original forecasts and contain the same columns as the original data.

#### Mean squared error (MSE) and mean absolute error (MAE).

MAE and MSE assess deviations in the mean model fit from the original time series data. Specifically, MAE is given by [78]:


$$\begin{array}{c} MAE=\frac{1}{N}\sum {\mathrm{\backslash nolimits}}_{h=1}^{N}\left|{\hat{y}}_{t}-y_{t}\right|, \end{array}$$ (16)

and MSE by [78]:


$$\begin{array}{c} MSE=\frac{1}{N}\sum {\mathrm{\backslash nolimits}}_{h=1}^{N}{\left({\hat{y}}_{t}-y_{t}\right)}^{2}. \end{array}$$ (17)

In both equations, $y_{t}$ is the original data of the process of interest at time t, and ${\hat{y}}_{t}$ is the model fit or forecasted value at time t. N represents the total length of time under evaluation (i.e., calibration or forecast period).

#### Prediction interval coverage (PI coverage) and weighted interval scores (WIS).

*StatModPredict* provides PI coverage and WIS as measures of a model’s fit and forecast uncertainty. PI coverage represents the proportion of observed data within the PI selected by the user. The WIS, described in [14], uses quantiles of the predictive forecast distribution, specifically by combining a set of interval scores (IS) obtained from probabilistic forecasts [79]. Only a central (1−α)*100% PI is needed to calculate an IS [79] as indicated below:


$${IS}_{\alpha }(F,y)=(u-l)+\frac{2}{\alpha }*(l-y)*1(y<l)+\frac{2}{\alpha }*(y-u)*1(y>u).$$ (18)

**1** is an indicator function, where 1(y < l= 1 if y < l and zero otherwise. The $\frac{\alpha }{2}$ and $1-\frac{\alpha }{2}$ quantiles of the forecast *F* are represented by *l* and *u*. The IS consists of three distinct quantities [79]:

1. The sharpness of *F*, given by the width *u* − *l* of the central (1 − α× 100%  PI.
2. A penalty term $\frac{2}{\alpha }*(l-y)*1(y<l)$ for the observations that fall below the lower end point *l* of the (1 − α× 100%  PI. This penalty term is directly proportional to the distance between *y* and the lower end *l* of the PI. The strength of the penalty depends on the level.
3. An analogous penalty term $\frac{2}{\alpha }*(y-u)*1(y>u)$ for the observations falling above the upper limit *u* of the PI.

As indicated in the “Quantile Forecast” box on the *Forecasting* page, *StatModPredict* provides multiple central PIs at different levels $\left(1\;-\;\alpha_{1}<\;(1\;-\;\alpha_{2})\;<\;\cdots \;<\;(1\;-\;\alpha_{k}\right)$, including the central prediction interval (m) at level $1\;-\;\alpha_{0}⟶\;0$ (first column of the box). From this, the WIS is calculated by [4,14,80]:


$$\begin{array}{c} {WIS}_{\alpha_{0:K}}(F,y)=\frac{1}{K+\frac{1}{2}}*(w_{0}*|y-m|+\sum {\mathrm{\backslash nolimits}}_{k=1}^{K}w_{k}*{IS}_{\alpha_{k}}(F,y)) \end{array}$$ (19)

where, $w_{k}=\frac{\alpha_{k}}{2}$ for *k* = 1,2, …. *K* and $w_{0}=\frac{1}{2}$.

#### Akaike Information Criterion (AIC).

The Akaike Information Criterion (AIC) is frequently used to select, evaluate, and compare model fits [81,82]. It is as follows [82]:


AIC=−2LL+(2k) (20)

where *LL* is the log-likelihood, and *k* represents the number of parameters included in the model. The AIC for the model fit is directly available as part of the *auto.arima()* function [45]; however, for the GLMs and GAMs, the AIC must be manually calculated using Eq. 20. *StatModPredict* obtains the log-likelihood directly from the GLM and GAM fits, utilizing the *logLik()* function [83]. The AIC only applies to frequentist methods, so the dashboard will not supply the value for the Prophet model, a Bayesian-like framework.

#### Corrected akaike information criterion (AICc).

The bias-corrected Akaike Information Criterion (AICc) is a variation of the AIC that adjusts for small sample sizes [82]. It is as follows:


$$AICc=-2LL+(2k)+\frac{2*k*(k+1)}{n-k-1}$$ (21)

where *LL* is the log-likelihood, *k* represents the number of parameters included in the model, and the calibration period length is given by *n*. Like AIC, the AICc for the model fit is directly available as part of the *auto.arima()* function [45] and must be manually calculated for the GLMs and GAMs. The dashboard utilizes the *logLik()* function to obtain the log-likelihood directly from the GLM and GAM fits [83]. The AICc only applies to frequentist methods, so the dashboard will not supply the value for the Prophet model, a Bayesian-like framework.

#### Bayesian information criterion (BIC).

The Bayesian information criterion (BIC), also known as Schwarz’s Bayesian criterion (SBC), is frequently applied in the model selection process. As with the AIC, the BIC is available for any model where the log-likelihood can be calculated [84]. It is given by the following:


BIC=−2LL+k*log(n) (22)

where *LL* is the log-likelihood, *k* represents the number of parameters included in the model, and the calibration period length is given by *n*. The BIC for the model fit is directly available as part of the *auto.arima()* function [45]. However, as above, *StatModPredict* manually calculates the BIC for the GLMs and GAMs and obtains the log-likelihood utilizing the *logLik()* function [83]. The BIC only applies to frequentist methods, so the dashboard will not supply the value for the Prophet model, a Bayesian-like framework.

#### Winkler and skill scores.

To facilitate model comparison, we also provide both crude and average Winkler and skill scores [62]. As with the other included metrics, “crude” refers to where one set of metrics (Winkler Scores or skill score) is available for each forecast conducted. “Average” scores refer to the average Winkler or skill score across forecast dates for each location (or group), model, and forecasting horizon. Both crude and average metrics are available for download as *.csv files.

Skills scores provide one method of calculating the proportion of improvement of one model over another model based upon MSE, MAE, WIS, and Winkler Scores (PI coverage). The formula is as follows [62]:


$$\frac{Baseline\;Model\;-\;Comparison\;Model}{Baseline\;Model}*100$$ (23)

The baseline model refers to the metric of the model that a user may want to compare the same metric for another model (i.e., comparison model) against. In place of PI coverage, the dashboard employs Winkler Scores in the skill scores calculations. Winkler scores are calculated as follows [62]:


$$\begin{array}{c} W_{\alpha ,t}=\left\{ \;\;\;\begin{array}{c} \left(u_{\alpha .t}-l_{\alpha ,t}\right)+\frac{2}{\alpha }\left(l_{\alpha ,t}-y_{t}\right),\;if\;y_{t}<l_{\alpha ,t}\\ \left(u_{\alpha .t}-l_{\alpha ,t}\right),\;if\;l_{\alpha ,t}\leq y_{t}\leq u_{\alpha ,t}\;\\ \left(u_{\alpha .t}-l_{\alpha ,t}\right)+\;\frac{2}{\alpha }\left(y_{t}-u_{\alpha ,t}\right)\;if\;y_{t}>u_{\alpha ,t}\;\\ \; \end{array}\right.\; \end{array}$$ (24)

where u and l refer to the upper and lower bounds of the selected PI interval at time t. The parameter $y_{t}$ represents the observed data at time t.

#### Available metrics output.

**Fig 5:**
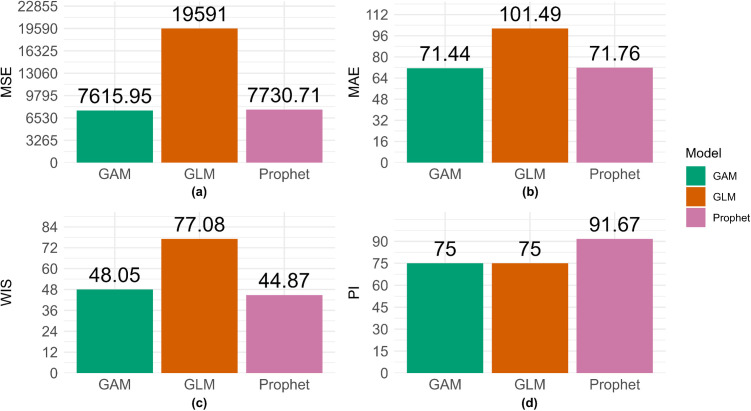
Model fit metrics obtained for the Northeast United States using a 12-year calibration period (2008-2019). This figure shows the mean squared error (MSE), mean absolute error (MAE), weighted interval score (WIS), and 95% prediction interval coverage (95% PI) metrics for the model fit of the GAM, GLM, and Prophet model. The associated fits were obtained utilizing 12 years of incident HIV diagnoses (2008 through 2019) obtained from the publicly available Centers for Disease Control and Prevention (CDC) *AtlasPlus* dashboard [55], and assuming the default settings of *StatModPredict*.

**Fig 6:**
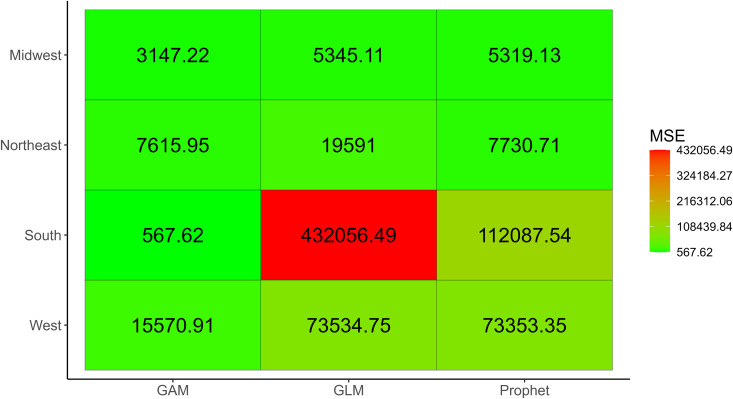
Average mean squared error for model fits obtained for four United States regions. This figure shows the average MSE across all selected forecast period dates (i.e., 2019) for all available locations (Midwest, Northeast, South, West). The associated fits were obtained utilizing 12 years of incident HIV diagnoses (2008 through 2019) obtained from the publicly available Centers for Disease Control and Prevention (CDC) *AtlasPlus* dashboard [55], and assuming the default settings of *StatModPredict*.

The dashboard provides crude metrics, where one set of metrics is available for each forecast conducted, and the average metrics. “Average” here refers to the performance metrics being averaged across forecast dates for each location (or group), model, forecasting horizon, and calibration period. Both the crude and average metrics are available for download as *.csv files. Users can also obtain visualizations of all performance metrics included in the dashboard, such as shown in Fig 5 for the crude performance metrics and Fig 6 for the average performance metrics. Please note that only the MSE, MAE, WIS, and PI coverage will be plotted for the crude metrics, and that *StatModPredict* does not provide visualizations for the results of the Ljung-Box test.

### Model comparison page

*StatModPredict* facilitates model-to-model comparisons by allowing users to input forecast and performance metrics files for “outside models” or models not included within the dashboard to obtain comprehensive visualizations and *.csv outputs. However, unlike other features of the dashboard, which provide flexibility in the file specifications, very specific file structures and naming conventions must be followed as discussed in detail below. All statistical model specifications and processing must be completed before proceeding with model comparison.

#### Preparing the forecast files.

The dashboard allows users to load multiple forecast files, which use the following naming scheme:

<Model Framework>-< Model>-horizon-<Horizon Number>-calibration-<Calibration Size>-<Location>-<Forecast Date>.csv.

Each entry in “<>” indicates file name elements specific to the forecast of interest. < Model Framework> refers to the overall framework or general model structure used, and <Model> refers to the specific model indicator or name. No restrictions exist on the nomenclature used for <Model Framework> or <Model > . < Horizon Number> is the length of the forecasting horizon, and <Calibration Size> is the length of the calibration period used to obtain the forecast. Regarding <Location>, it must match one of the locations (or groups), including in capitalization, utilized in the previously loaded time series. Finally, < Forecast Date> is the last data date used to calibrate the model. If working with yearly data, the <Forecast Date> needs to be a four-digit year (“YYYY”), a number if employing a time index, or “MM-DD-YYYY” format if working with weekly or daily data.

**Fig 7:**
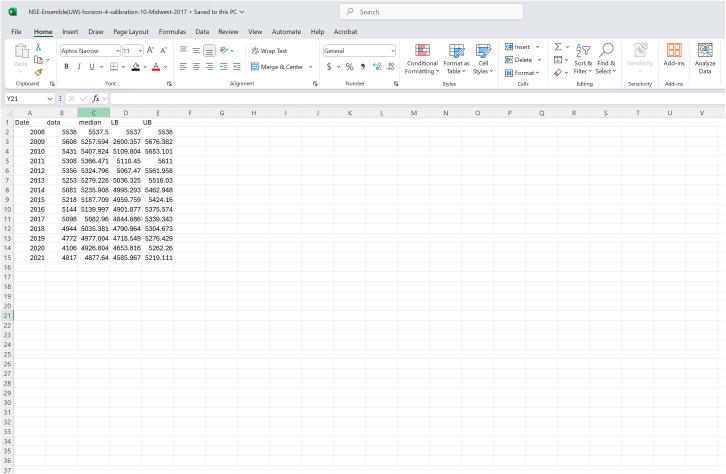
A screenshot of the required file structure for an “outside model” forecast. The file shows a forecast produced using the *n*-sub-epidemic framework, a MATLAB-based modeling tool [14]. As can be seen in the image, the file must contain five columns: “Date”, “data”, “median”, “LB”, and “UB”.

The file structure format must match that used for the “Formatted Forecasts” on the *Forecasting* page. Therefore, the column names need to include: “Date,” “data,” “median,” “LB,” and “UB,” maintaining the same capitalization and order presented in Fig 7.

“Date” refers to the respective time point the row of data are associated with. If working with daily or weekly temporal resolutions, the date must be formatted in “YYYY-MM-DD” format; yearly data are “YYYY”. When a time index represents the temporal resolution, it follows the same convention discussed for the input data above. The “data” column refers to the observed values at each time point, “median” is the fit or forecasted value, and “LB” and “UB” refer to the upper and lower bounds of the selected prediction interval.

#### Preparing the performance metrics files.

*StatModPredict* also allows users to load multiple “outside” performance metrics. Each forecast file must include a *.csv extension and use the following naming scheme:

Performance-<Type>-horizon-<Horizon Number>-calibration-<Calibration Size>.

<Type> refers to the type of performance metrics shown in the file; either “Fit” for model fit metrics or “Forecast” for forecast performance metrics. <Horizon Number> is the length of the forecasting horizon, and <Calibration Size> refers to the length of the data used to calibrate the model.

**Fig 8:**
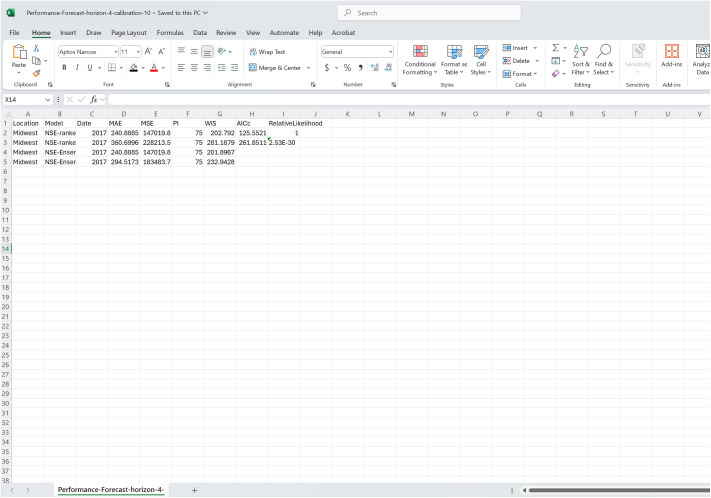
A screenshot of the required structure for an “outside model” performance metrics file. The file shows multiple model performance metrics corresponding to forecasts produced using the *n*-sub-epidemic framework (NSE-Ensemble), a MATLAB-based modeling tool [14]. At a minimum, the file must contain the “Location”, “Model”, and “Date” columns. The remaining columns correspond to the available performance metrics. If a given metric, such as the AICc and Relative Likelihood, for the NSE-Ensemble or other “outside” models is unavailable, the associated cell should be left blank.

As shown in Fig 8, the performance metrics files must contain at least three columns, “Location”, “Model”, and “Date”. The remaining columns correspond to unique statistics available for the given performance metric type (“Fit” or “Forecast”), forecasting horizon, and calibration period length. The associated cell should be left blank if a given metric is unavailable for a location, model, and date combination. Additionally, if including the PI coverage for an outside model, the label “PI” must be used to ensure correct merging with the metrics for the dashboard models.

“Location” is the given geographical space or grouping that the metrics correspond to. It must match one of the locations or groups, including in capitalization, used in the data employed throughout the rest of the dashboard. The “Model” is the label from which the metrics were derived for the modeling framework. “Date” refers to the respective time point the row of data are associated with. If working with daily or weekly data, the date must be formatted in “YYYY-MM-DD” format; yearly data are “YYYY”. When a time index represents the temporal resolution, it follows the same convention discussed for the input data.

#### Available outputs.

After correctly formatting and naming the files following the description provided above, users can then read either type of file into the dashboard. If the user selects forecast files, *StatModPredict* will return individual figures for the input files and panel images containing all models (i.e., outside and dashboard) grouped by forecast date, location (or group), calibration period, and forecasting horizon. Users who select performance metrics files will receive the same information produced within the *Model Metrics* page; however, it will now contain metrics related to the outside models. Additional examples of the produced output can be found in the case study presented below.

## Case study: The ongoing HIV epidemic in the United States

To illustrate the real-world functionality of *StatModPredict*, we will use publicly available, yearly, aggregated and de-identified incident HIV diagnoses data (2008–2019) for the United States obtained from the publicly available CDC’s *AtlasPlus* dashboard [55]. The time series consists of five columns: Year, Midwest, Northeast, South, and West. Column one contains the years of data included within the time series, and the remaining columns include yearly counts of incident HIV diagnoses for four regions of the United States. To maintain simplicity, we will only be working with the Midwest data for this example. However, *StatModPredict* does allow for multiple locations or groupings of data (i.e., non-date columns) to be selected at once. The tutorial data are available in the associated GitHub repository [54], with its layout visualized in Fig 1.

### Forecasting parameters

**Fig 9:**
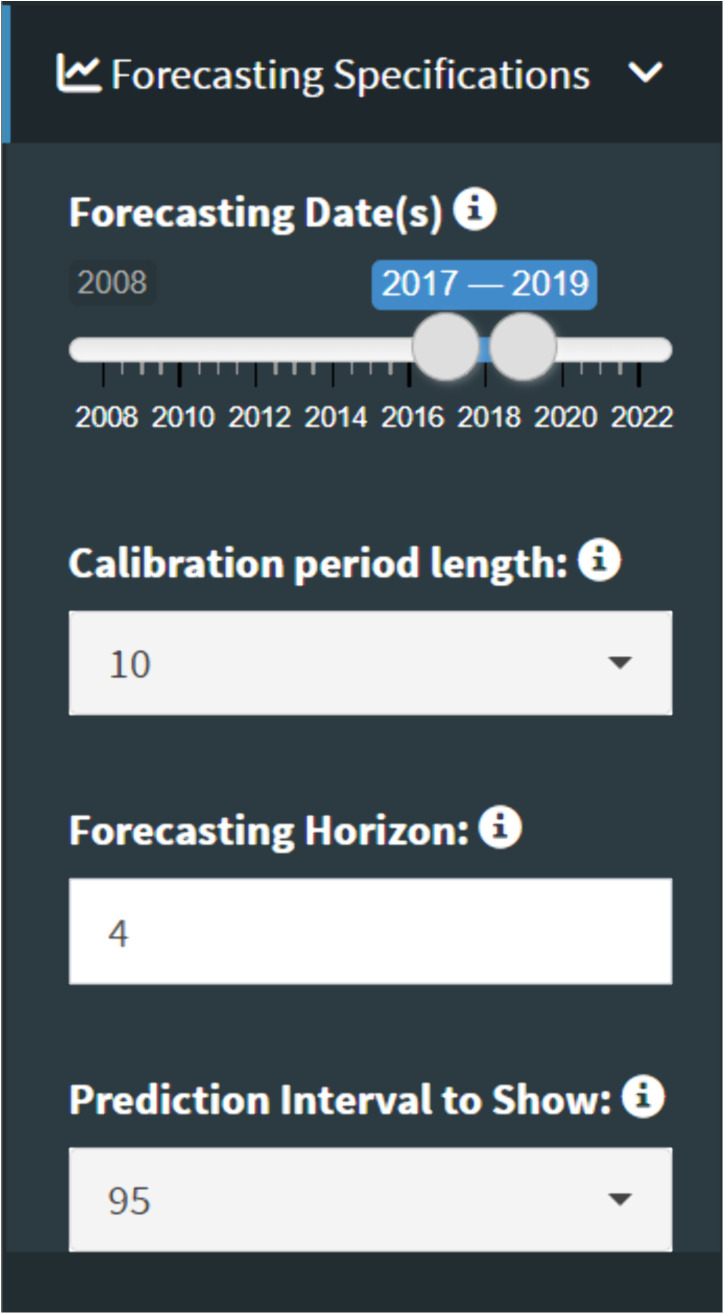
The forecasting specifications utilized in the tutorial. As multiple forecast dates have been selected, multiple forecasts will be produced using 10-year calibration periods and forecasting four years into the future.

After loading the time series and selecting the Midwest location, we can indicate our desired forecast dates, calibration period length, forecasting horizon, and PI. This tutorial will use forecast dates from 2017–2019, 10-year calibration periods, a 4-year forecasting horizon, and 95% PIs (Fig 9). Therefore, *StatModPredict* will conduct three forecasts using data from 2007 to 2017, 2008–2018, and 2009–2019. With a forecasting horizon set to 4 years, we will receive forecasts from the forecast date (i.e., last date of the calibration period), 4 years into the future. For example, for a forecast date of 2019, our projections will cover 2020–2024.

Finally, we select our models (ARIMA, GLM, GAM, and Prophet) and specify their parameters. For this tutorial, we employ the auto-populated baseline model customizations for all four models in the dashboard. However, these parameters and other options can be adjusted further as needed.

### Fitting and forecasting

**Fig 10:**
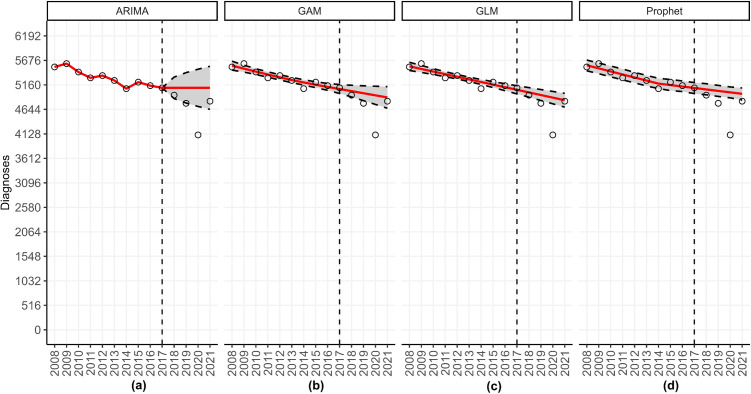
The forecast panel illustrating HIV forecasts for the Midwest United States using data through 2017. This figure shows the forecasts produced for the Midwest United States utilizing yearly incident HIV diagnoses obtained from the publicly available Centers for Disease Control and Prevention (CDC) *AtlasPlus* dashboard [55], and assuming the default settings of *StatModPredict*. It can be obtained from the top box of the *Forecasting* page by clicking the “See Panels” option. For each model, the grey region corresponds to the selected prediction interval, the vertical dashed line is the forecast date (i.e., the last date used to calibrate the model), the red line is the estimated fit or forecast, and the open circles are the observed data. As can be seen, the ARIMA model forecasted a steadying of cases, whereas the other three models predicted a slight decrease in the number of HIV diagnoses through 2021. However, all four models over-predicted the number of incident diagnoses at all points of the forecast period.

After the models have concluded running, the associated output will be visible on the main body of the dashboard. The top panel of the dashboard shows the individual forecast figures (i.e., one model at a time) and the panels of forecast figures for the Midwest, grouped by forecast date and calibration period length (Fig 10), and the underlying data files.

**Fig 11:**
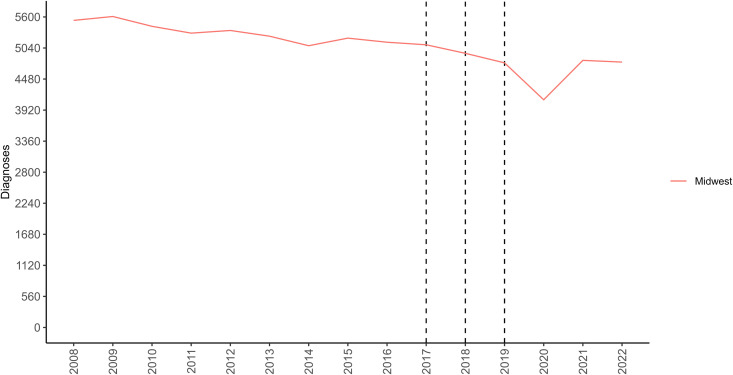
The time series illustrating the trajectory of incident HIV diagnoses for the Midwest United States. The colored line corresponds to the respective time series data, and the vertical dashed lines indicate the forecast dates selected in the sidebar panel.

In addition to obtaining the forecast figures and their underlying data, we can also get 22 different quantiles, including the model fit and forecasted values for the Midwest and each selected combination of model and calibration period, and a customizable time series figure visualizing the trajectory of incident HIV diagnoses in the region (Fig 11).

### Evaluating model fit and forecast performance

Having run our forecasts, we can now obtain model fit and forecasting performance metrics. The metrics are automatically calculated once *StatModPredict* has completed all necessary forecasting procedures using the incident HIV diagnoses data. During this process, multiple outputs become available, including the average metrics (i.e., averaged across forecast dates), crude metrics (i.e., one set for each forecast), related visuals, Winkler Scores, and skill scores.

**Fig 12:**
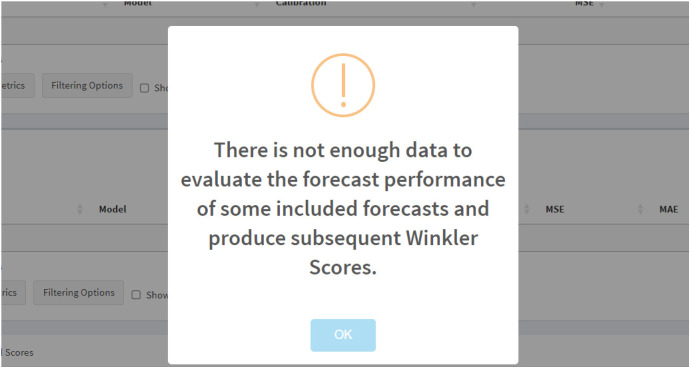
The warning that appears when insufficient data are available to calculate forecast performance metrics. This warning appears when users navigate to the *Forecasts* page via the “Select metrics to show” option. This warning tells the user that some or all forecast performance metrics cannot be calculated.

As discussed above, forecast performance metrics are only available when the data exists to compare the forecast against. In our example, the 2019 forecast date produced a forecast through 2024, a year for which we do not have data available. Therefore, when navigating to the *Forecasts* page via the “Select metrics to show” option, the following warning appears (Fig 12).

Nevertheless, the performance metrics for both the 2017 and 2018 forecast dates are available.

**Fig 13:**
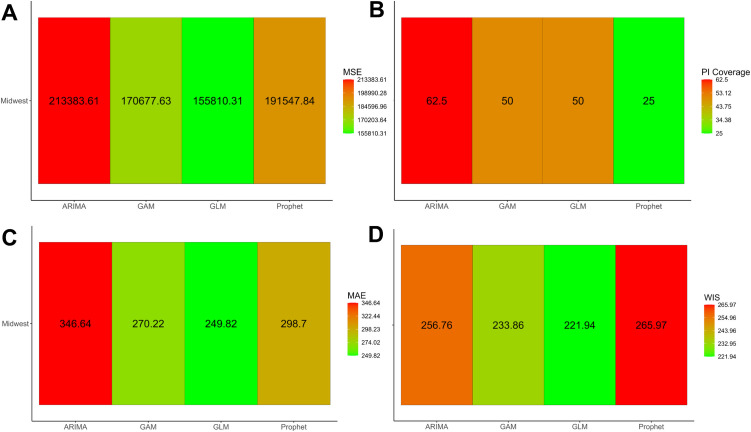
Average forecast performance metrics for the Midwest United States. The above figure provides the average mean squared error (A), 95% PI coverage (B), mean absolute error (C), and weighted interval score (D) obtained from the forecast produced with a 2017 and 2018 forecast date. For both dates, the previous 10 years were used to calibrate the model, and the forecasts were produced 4 years out. Overall, it appears that the GLM performed best in forecasting the trajectory of HIV diagnoses in the Midwest United States.

Overall, the ARIMA model best fits the data based on the average AICc, AIC, and BIC across forecast dates, compared to the GAM and GLM. However, the Prophet model produced a better fit compared to the GLM and GAM according to the average MSE, 95% PI, and WIS (Table 2). Regarding forecasting performance, the GLM produced the best average (2017 & 2018) MSE, MAE, and WIS compared to the other three models (Fig 13).

**Table 2:** The average model fit metrics for three forecast dates.

Model	MAE ^a^	MSE ^b^	95% PI ^c^	WIS ^d^	AICc ^e^	AIC ^f^	BIC ^g^
ARIMA^h^	–	–	–	–	114.13	112.61	112.93
GLM ^i^	48.39	3615.56	76.67	32.98	126.53	117.81	118.99
GAM ^j^	56.79	4827.68	60	40.6	123.18	119.18	120.09
Prophet ^k^	48.78	3508.18	93.33	30.16	–	–	–

The statistics contained in this table correspond to the average model fit performance metrics averaged across the 2017, 2018, and 2019 forecast dates. The models were fit to 10 years of HIV diagnoses data for the Midwest United States.

^a^Mean Absolute Error;

^b^Mean Squared Error;

^c^95% Prediction Interval Coverage;

^d^Weighted Interval Score;

^e^Corrected Akaike Information Criterion;

^f^Akaike Information Criterion;

^g^Bayesian Information Criterion;

^h^Auto-regressive integrated moving average;

^i^Generalized linear model;

^j^Generalized additive model;

^k^Meta (Facebook’s) Prophet model.

*Some model fit metrics cannot be calculated for the ARIMA-type models due to the structure of the *auto.arima()* function. Additionally, as the Prophet model is a Bayesian-like Framework, AICc, AIC, and BIC cannot be calculated. The missing values are represented by “-” in the table.

### Model comparison

**Fig 14:**
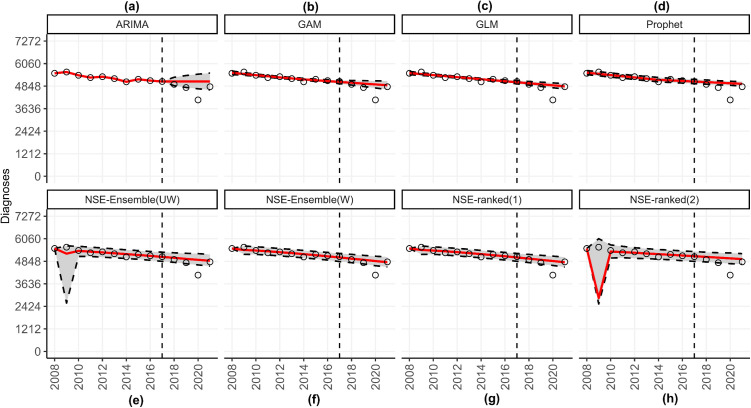
A forecast panel illustrating HIV forecasts in the Midwest US for eight models through 2021. The following figure can be obtained from the top portion of the *Model Comparison* page. For each model, the grey region corresponds to the selected prediction interval (95% PI for this example), the vertical dashed line is the forecast date (i.e., the last date used to calibrate the model), the red line is the estimated fit or forecast, and the open circles are the observed data.

For this tutorial, we compare the ARIMA, GLM, GAM, and Prophet model against the top-ranked, second-ranked, weighted, and unweighted ensemble models obtained from the *n*-sub-epidemic framework [14] for the Midwest using a forecast date of 2017. The *n*-sub-epidemic framework is available as part of the *SubEpiPredict* MATLAB toolbox [14]; it is not included as part of the *StatModPredict* dashboard. Additional details about the framework can be found in Ref. [14]. After preparing the *n*-sub-epidemic forecast and performance metrics files following the above format, we can load them into the dashboard via their respective upload buttons. We visually compare the associated model fits and subsequent forecasts for the Midwest United States in Fig 14.

Additionally, we can obtain both the crude and average metrics figures as before, and the performance metrics combined into a single table. For example, Table 3 includes the forecast performance metrics for all dashboard and *n*-sub-epidemic framework models. Overall, it appears that the *n*-sub-epidemic weighted ensemble model outperformed the dashboard models across all metrics using a forecast date of 2017, a calibration period of 10 years, and forecasting 4 years into the future (Table 3).

**Table 3:** A comparison of model forecasting metrics across all four models included within the dashboard, and for the top-ranked, second-ranked, weighted, and unweighted ensemble *n*-sub-epidemic models.

Model	MAE ^a^	MSE ^b^	95% PI ^c^	WIS ^d^
ARIMA ^e^	438.25	298254.25	50	323.23
GLM ^f^	259.52	162937.91	50	228.97
GAM ^g^	302.67	188638.25	50	251.21
Prophet ^h^	358.1	227545.44	0	313.54
NSE 1^st^ Ranked ^i^	**240.89**	**147019.77**	**75**	202.79
NSE 2^nd^ Ranked ^j^	360.7	228213.53	75	281.19
W – NSE Ensemble ^k^	**240.89**	**147019.77**	**75**	**201.9**
UW – NSE Ensemble ^l^	294.52	183483.69	75	232.94

The statistics contained in this table correspond to the crude forecast performance metrics for the dashboard and *n*-sub-epidemic framework models. All models were fit to data from the Midwest, used a forecast date of 2017, were calibrated with 10 years of data, and forecasted four years out.

^a^Mean Absolute Error;

^b^Mean Squared Error;

^c^95% Prediction Interval Coverage;

^d^Weighted Interval Score;

^e^Auto-regressive integrated moving average;

^f^Generalized linear model;

^g^Generalized additive model;

^h^Meta (Facebook’s) Prophet model;

^i^*n*-sub-epidemic 1^st^ Ranked model;

^j^*n*-sub-epidemic 2^nd^ Ranked model;

^k^Weighted ensemble *n*-sub-epidemic model;

^l^Unweighted ensemble *n*-sub-epidemic model.

*Bold values indicate the best-performing models for the given metric.

## Conclusions

Infectious disease forecasting provides critical insights into the potential trajectory of epidemics, resource allocation needs, and policy shifts. Given the discipline’s recent expansion, equipping future and early-career professionals with a strong foundation in established modeling techniques is essential to advancing the field. However, existing forecasting tools’ programming requirements, paid subscriptions, or lack of analysis flexibility (i.e., model and parameter selection) can hinder both student engagement and real-time use by public health professionals and policymakers. As discussed above, *StatModPredict* provides a robust and flexible graphical interface for fitting and forecasting with four established statistical models, evaluating their fits and subsequent forecasts, and facilitating comparisons with other models. Therefore, its uniqueness lies in its ability to provide a graphical interface with an end-to-end workflow for real-time and retrospective forecasting not readily available with other identified software.

Although *StatModPredict* aims to improve accessibility to statistical forecasting, it is not without limitations. *StatModPredict* assumes users have a basic understanding of forecasting processes and the included statistical methods. The interface does not provide any specific modeling or forecasting guidance, apart from brief commentary on the appropriate data for each statistical model included above. Ultimately, the primary purpose of *StatModPredict* is to present a programming-free environment to interact with and visualize the results of ARIMA, GLM, GAM, and Prophet forecasting analyses. *StatModPredict* is built entirely in R and is dependent on multiple R packages. Therefore, any updates to existing packages or software may result in a breakdown of the interface’s functionality. We frequently check and update *StatModPredict* to ensure it is compatible with the most up-to-date versions of R, RStudio, and related packages. Additionally, we host the dashboard within a GitHub repository [54], allowing for indexed updates and reporting of any potential issues. Our interface utilizes existing packages for the ARIMA [45], GLM [46,47], GAM [48], and Prophet [49] models; therefore, any limitations associated with their corresponding R-packages and underlying models extend to *StatModPredict*. Finally, the dashboard can only be run locally. Future iterations of *StatModPredict* will include the development of a CRAN package, such as *predictoR()* [32] and *Greymodels()* [31], and a web-based interface to increase dashboard accessibility.

The flexibility in *StatModPredict*’s required data structure and model specification process extends its potential application to other disciplines (e.g., business and weather), where the included models are often employed [57–61,72,73,85–89]. Nevertheless, future updates of *StatModPredict* may include additional features such as more available evaluation statistics for each model, the ability to run multiple forecasting horizons simultaneously, and incorporating an “ensemble” feature [4,14]. Such updates to the interface’s flexibility would increase its utility for both its intended audience and others looking to conduct time series forecasting.

Overall, *StatModPredict* offers an interactive and intuitive environment for exploring and applying foundational statistical forecasting methods, suitable for individuals with varying programming experience. We encourage all interested users, including students and professionals, to engage with the interface and provide feedback to support its ongoing development.

## Supporting information

S1 File The *StatModPredict* R-Shiny application code. S1 File contains the most up-to-date version of *StatModPredict* Shiny application as of *July 14, 2025*.
(ZIP)
